# The fate of a designed protein corona on nanoparticles in vitro and in vivo

**DOI:** 10.3762/bjnano.6.5

**Published:** 2015-01-06

**Authors:** Denise Bargheer, Julius Nielsen, Gabriella Gébel, Markus Heine, Sunhild C Salmen, Roland Stauber, Horst Weller, Joerg Heeren, Peter Nielsen

**Affiliations:** 1 Department of Biochemistry and Molecular Cell Biology, University Medical Center Hamburg-Eppendorf, Martinistr. 52, 20246 Hamburg, Germany; 2 Institute of Physical Chemistry, University Hamburg, Grindelallee 117, 20146 Hamburg, Germany; 3 Molecular and Cellular Oncology, ENT/University Medical Center Mainz, Langenbeckstr. 1, 55101 Mainz, Germany

**Keywords:** albumin, ^59^Fe, ^125^I, organ uptake, protein corona, SPIOs, transferrin

## Abstract

A variety of monodisperse superparamagnetic iron oxide particles (SPIOs) was designed in which the surface was modified by PEGylation with mono- or bifunctional poly(ethylene oxide)amines (PEG). Using ^125^I-labeled test proteins (transferrin, albumin), the binding and exchange of corona proteins was studied first in vitro. Incubation with ^125^I-transferrin showed that with increasing grade of PEGylation the binding was substantially diminished without a difference between simply adsorbed and covalently bound protein. However, after incubation with excess albumin and subsequently whole plasma, transferrin from the preformed transferrin corona was more and more lost from SPIOs in the case of adsorbed proteins. If non-labeled transferrin was used as preformed corona and excess ^125^I-labeled albumin was added to the reaction mixtures with different SPIOs, a substantial amount of label was bound to the particles with initially adsorbed transferrin but little or even zero with covalently bound transferrin. These in vitro experiments show a clear difference in the stability of a preformed hard corona with adsorbed or covalently bound protein. This difference seems, however, to be of minor importance in vivo when polymer-coated ^59^Fe-SPIOs with adsorbed or covalently bound ^125^I-labeled mouse transferrin were injected intravenously in mice. With both protein coronae the ^59^Fe/^125^I-labelled particles were cleared from the blood stream within 30 min and appeared in the liver and spleen to a large extent (>90%). In addition, after 2 h already half of the ^125^I-labeled transferrin from both nanodevices was recycled back into the plasma and into tissue. This study confirms that adsorbed transferrin from a preformed protein corona is efficiently taken up by cells. It is also highlighted that a radiolabelling technique described in this study may be of value to investigate the role of protein corona formation in vivo for the respective nanoparticle uptake.

## Introduction

Nanoparticles (NPs) have unique capabilities to interact with cells and organs which mark them as attractive working material in nanobioscience and nanomedicine. In order to make full use of their potential it is essential to understand what controls at the molecular level recognition by cells, cell entering and intracellular processing. Physicochemical properties of NPs such as material composition, size, shape, charge, and surface chemistry, have been reported to play signiﬁcant roles [1–7]. A small size, neutral or negative zeta potential, and extended PEGylation of the surface material are correlated with increased circulation time in blood after intravenous (i.v.) injection [4,6]. One important implication is that NP upon contact with biological matrices such as the blood, are immediately coated by a layer of proteins, resulting in a so called „protein corona“ [8–14]. The protein corona modifies and shields the surface of the xenobiotic particles and may subsequently influence or even determine their biological properties, and with this may influence their behavior in the microenvironment, i.e., their interaction with cells and tissues. Previous experimental studies have provided much insight in the layer thickness, composition of the protein corona, and the adsorption kinetics under different experimental setups. Various techniques such as ITC (isothermal titration calorimetry), SPR (surface plasmon resonance), DCS (differential centrifugal sedimentation), QCM (quartz crystal microbalance), and FCS (ﬂuorescence correlation spectroscopy) have been used to monitor the affinities of proteins for nanoparticles [15–20]. From FCS adsorption curves, Milani et al. showed the build-up of a strongly bound monolayer up to the point of monolayer saturation followed by a secondary, weakly bound layer [21]. This would confirm a longer discussed view of a first protein layer that interacts directly with the nanomaterial surface and is therefore tightly bound (hard corona), and a secondary layer (soft corona) that interacts with a weak protein–protein binding and exhibits dynamic exchange, if competing protein is added [8–11]. It is also reported that in the presence of plasma proteins, the hard corona is stable and retained on the nanoparticles as they enter cells and are trafficked to the lysosomes [22]. Recently, the protein corona formation in vitro has been found to mask transferrin conjugated with nanoparticles, and subsequently cause the loss of the designed function in transferrin receptor binding on the surface of cells [23].

It should be noted that most of the mentioned work was performed in in vitro experiments using partly also cell culture models. To date, much less information is available on the consequences of protein adsorption on nanoparticles in vivo. This is not a trivial objection because the in vivo situation is much more complex. After intravenous injection in a living organism nanoparticles are immediately swirled around with thousands of plasma proteins throughout the blood vessel system, are facing billions of moving cells and the large surface area of vascular endothelia cells.

The aim of the present study is to reexamine the concepts of protein corona formation in vitro and in vivo using ^59^Fe-radiolabeled nanoparticle cores and ^125^I-labelled model proteins. It is awaited that this technique is of special value to quantify the distribution and fate of functionalized nanoparticles also in vivo.

## Results

### Particle synthesis and characterization

**Figure 1:**
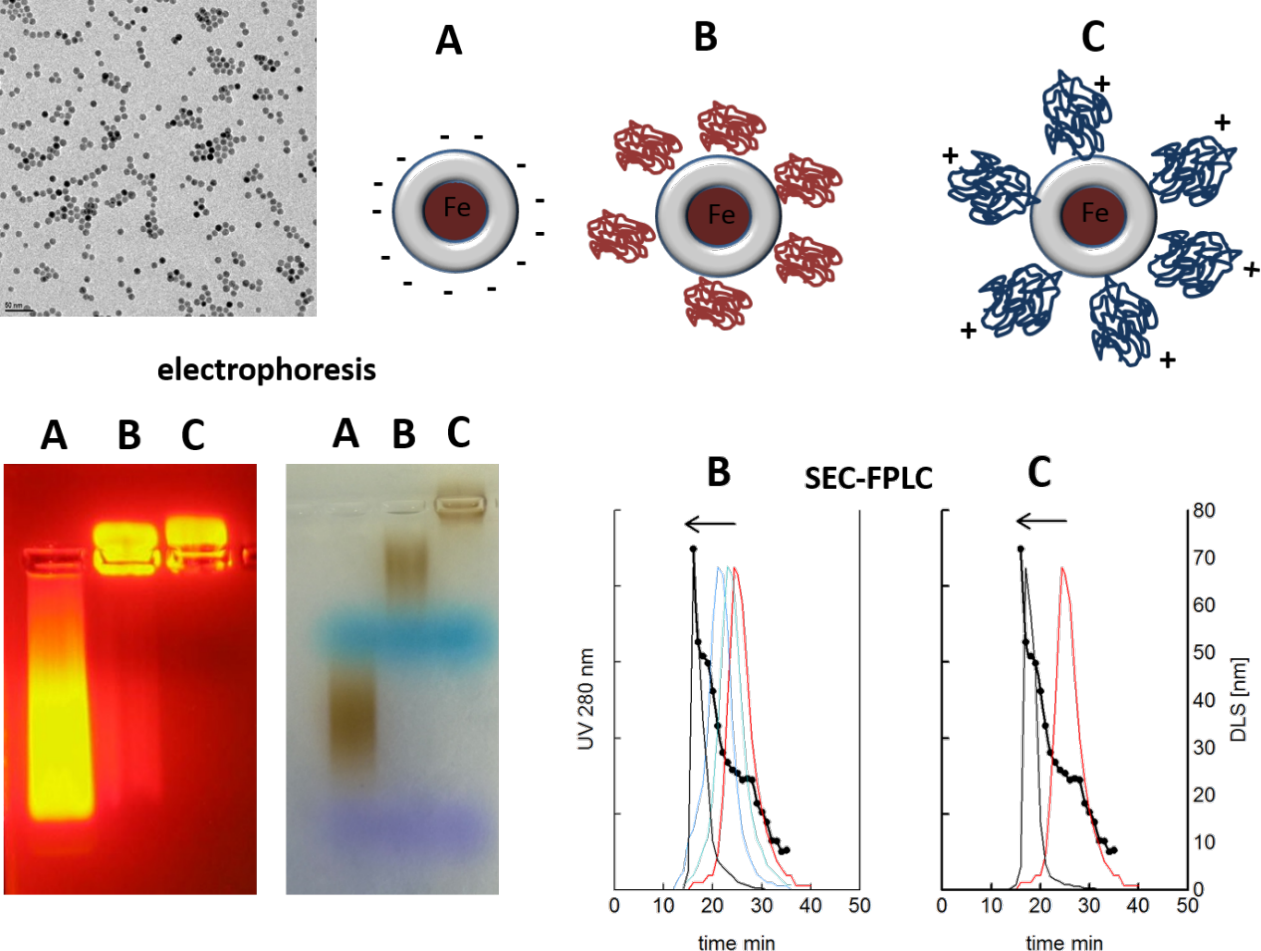
Synthesis and characterization of polymer-coated SPIOs with different surface charge due to PEGylation with mono- or bifunctional PEGs. Monodisperse oleic acid stabilized iron oxide cores (11 nm iron oxide core, see electron micrograph) were used as starting material. Whereas our polymer coated model SPIOs (A) is negatively charged due to free carboxyl groups (25 nm, hydrodynamic diameter), reaction with methoxy-PEG amine resulted in a more neutral particle (B), reaction with PEG-bisamine in an even cationic particle (C) as seen in electrophoresis (left Quantum dots, right SPIOs with the same polymer-coating and the same pegylation). Modification of the EDC concentration resulted in gradually PEGylated products, which can be detected by increasing size (arrows) in size-exclusion-FPLC and DLS. The FPLC was calibrated with human plasma by DLS-analysis of proteins in collected fractions (closed circles).

Radiolabelling of both the nanoparticle cores and of adsorbed proteins offers a way to follow and quantify the fate of a designed protein corona not only in vitro but also in vivo*.* For this purpose, we used as model hydrophobic monodisperse iron oxide nanoparticles, obtained from a high-temperature synthesis, which were transferred into aqueous medium by encapsulation with the well-characterized amphiphilic polymer, poly(maleic anhydride-alt-1-octadecene) [24–25]. These particles are negatively charged due to the formation of carboxyl groups at the surface. To get a platform of particles with different surface characteristics we then used a poly(ethylenglycol)(PEG)-amine (C-PEG) or a PEG-α,ω-bisamine (N-PEG) in the presence of the coupling agent, 1-ethyl-3-(3-dimethylaminopropyl)carbodiimide (EDC) to covalently bind PEG to the particles which diminished or even reversed the charge as seen in electrophoresis (Figure 1) [26]. By modifying the EDC concentration, partly or completely PEGylated species could be obtained. Size exclusion chromatography and DLS showed the increase of the size, electrophoresis the change in charge of the particles (Figure 1).

### In vitro experiments

For in vitro experiments, a selection of these nanoparticles was incubated first with the test protein transferrin to perform a corona which was then replaced by albumin or plasma proteins. The adsorbed corona was compared in these experiments with covalently bound transferrin, induced by EDC coupling.

To quantify the binding or removal of proteins, transferrin or albumin were radiolabelled with ^125^I and incubated with the respective SPIO for 2 h at room temperature. In a first experiment, we incubated human ^125^I transferrin with a variety of C-PEG-SPIOs. Using a 100,000 Da filtration system, unbound free transferrin was removed and an aliquot was measured for γ-counts (Table 1).

**Table 1:** Binding of ^125^I-transferrin to different PEGylated SPIOs. C0.2K denotes a partly PEGylated SPIO with EDC in the synthesis (SPIO:EDC = 1:200); C10K, a fully PEGylated SPIO with SPIO:EDC 1:10000. +, EDC present in the initial transferrin coupling (SPIOs:EDC 1:1000); −, adsorbed transferrin with no EDC present.

	bound ^125^I-transferrin (%)	remaining ^125^I on particles after incubation with albumin (pmc-SPIOs+ = 100 %)	remaining ^125^I on particles after incubation with serum (pmc-SPIOs+ = 100 %)

pmc-SPIO−	78	85	89
pmc-SPIO+	76	100	100
C0.2K−	51	28	29
C0.2K+	54	31	44
C1K−	50	21	21
C1K+	51	92	99
C10K−	31	10	11
C10K+	46	51	58

**Figure 2:**
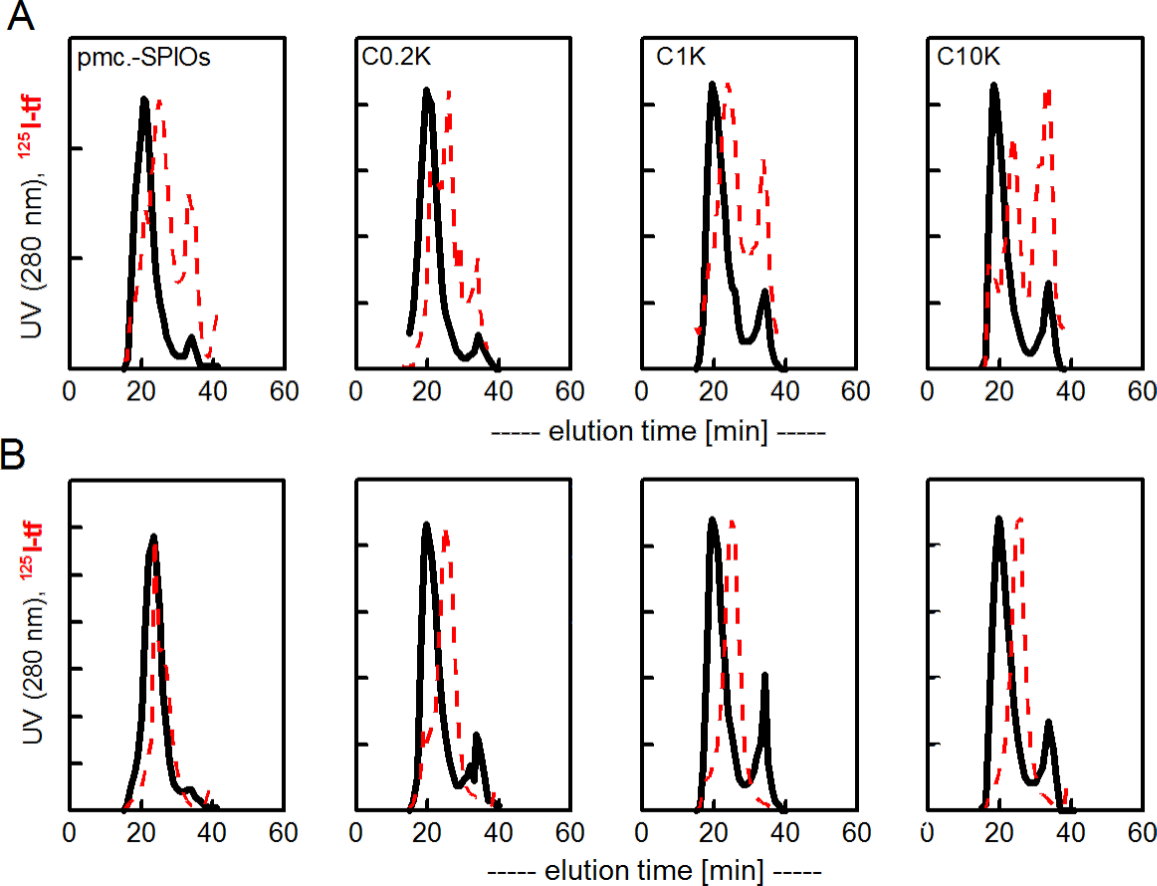
Stability of a preformed ^125^I-transferrin corona after exchange with excess of albumin. A, ^125^I-transferrin was first adsorbed to different PEGylated SPIOs. After removal of free unbound protein by filtration, a 3 fold excess of bovine albumin was added. FPLC analysis followed a 2 h incubation at room temperature. B, same experiment as in A but ^125^I-transferrin was covalently bound (EDC-coupling). A clear leakage of protein from the corona was observed when ^125^I-transferrin was initially adsorbed (upper lane) but not when covalently bound (bottom lane). ^125^I-activity in arbitrary units with a clearly less remaining activity with adsorbed transferrin than with covalently bound (see also Table 1). Filled lines, UV 280 nm; dashed lines, ^125^I-activity in fractions. The peak at 33 min represents free albumin or transferrin. FPLC analyses showed that the primarily adsorbed transferrin is substantially exchanged by albumin, whereas the covalently bound co-elutes only with the SPIOs and not with the peak of free protein. Finally, an excess of whole plasma was added to the reaction mixture under the same experimental conditions (Figure 3).

For the different SPIOs, the measured ^125^I-activity coeluted exclusively with the SPIOs-peak (data not shown), however, the amount of bound proteins was diminished with the grade of PEGylation with no observable differences between adsorbed and covalently bound transferrin. After addition of a 3 fold excess of unlabeled bovine albumin, the same procedure of ultrafiltration and SEC-FPLC was performed (Figure 2). Again, a leakage of the adsorbed but not the covalently bound transferrin was monitored which eluted in the FPLC as free protein.

**Figure 3:**
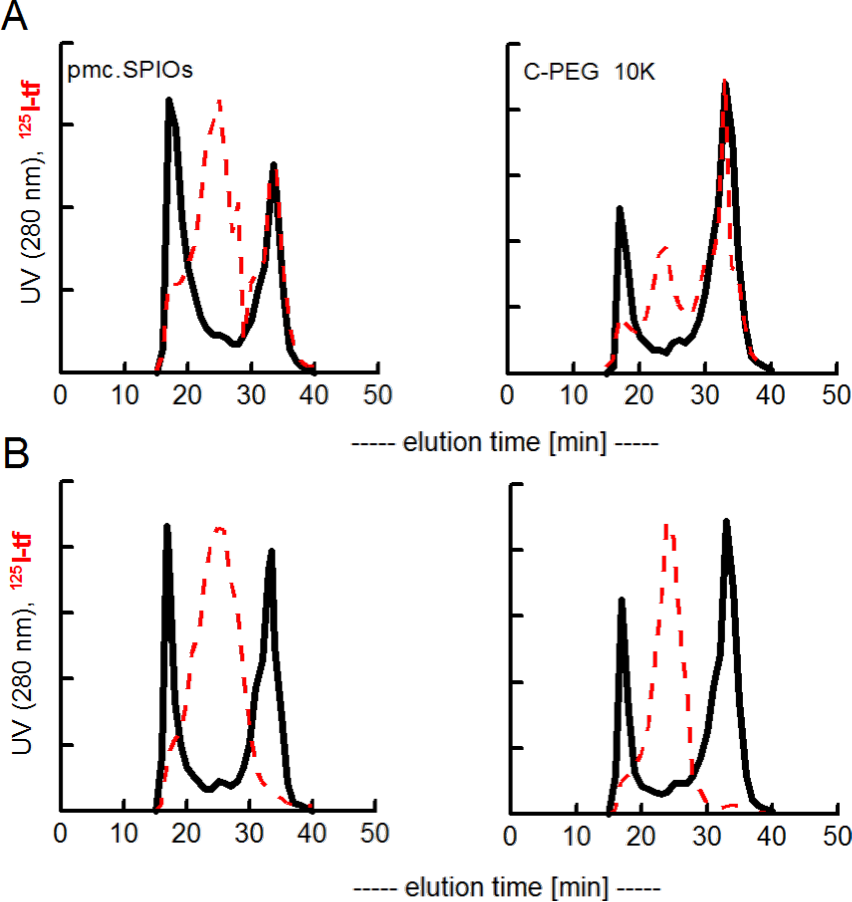
FPLC analysis of remaining transferrin after exchange with additionally added whole plasma (3 fold excess to initial transferrin). Same experiment as described in Figure 2. A, initial transferrin adsorbed; B, initial transferrin covalently bound. The peak at 33 min represents proteins with the size of transferrin (DLS: 9 nm).

**Figure 4:**
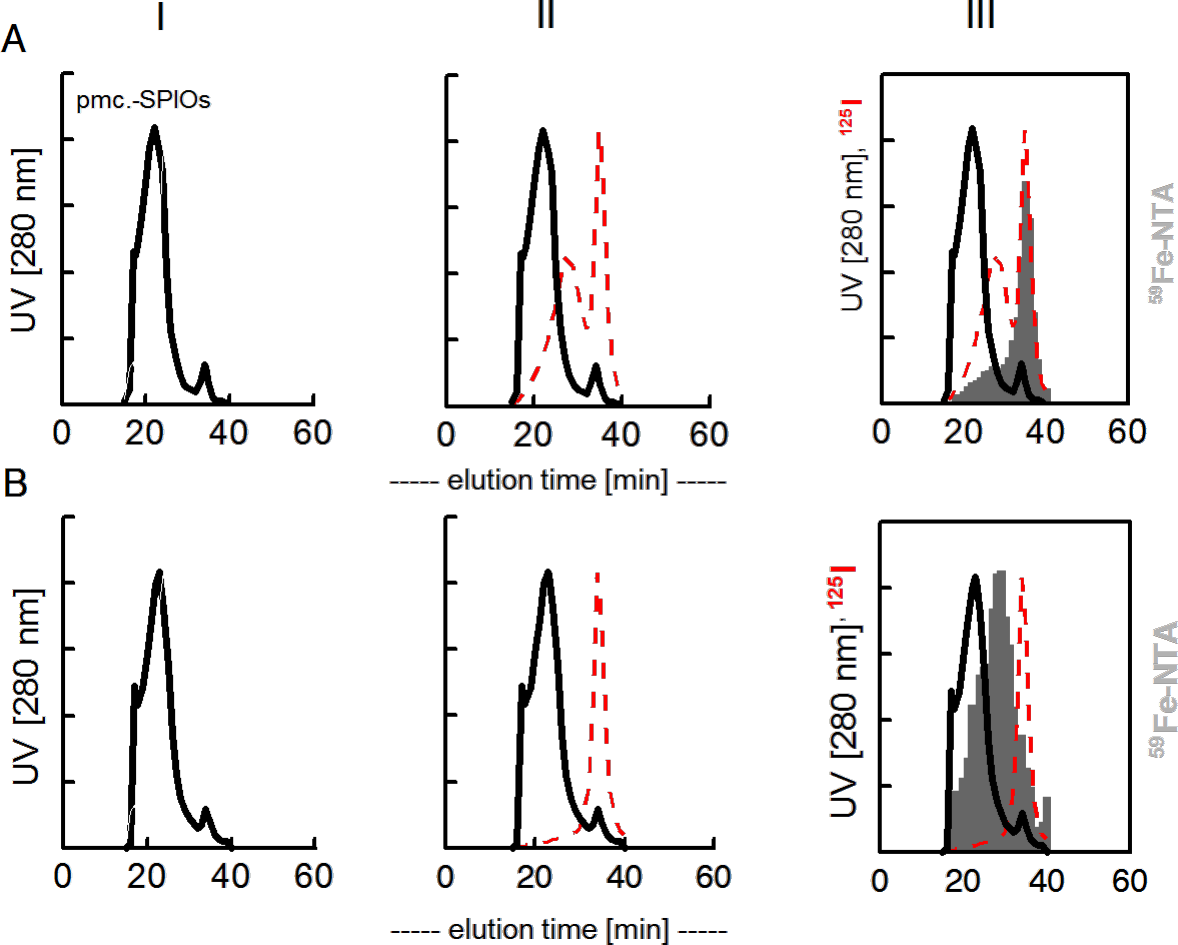
Stability of transferrin from a preformed corona on the polymer-coated, negatively charged nanoparticle. FPLC-chromatograms: I, upon transferrin binding the SPIOs with a hydrodynamic diameter of 25 nm increased the volume to about a diameter of 35 nm; II, a second incubation with ^125^I-albumin resulted in additional binding of ^125^I-albumin, III, labelling of transferrin on the SPIOs by incubation with ^59^Fe-NTA (filled area). A, initial transferrin adsorbed; B, initial transferrin covalently bound. The peak at 33 min represents proteins with the size of transferrin (DLS: 9 nm).

**Figure 5:**
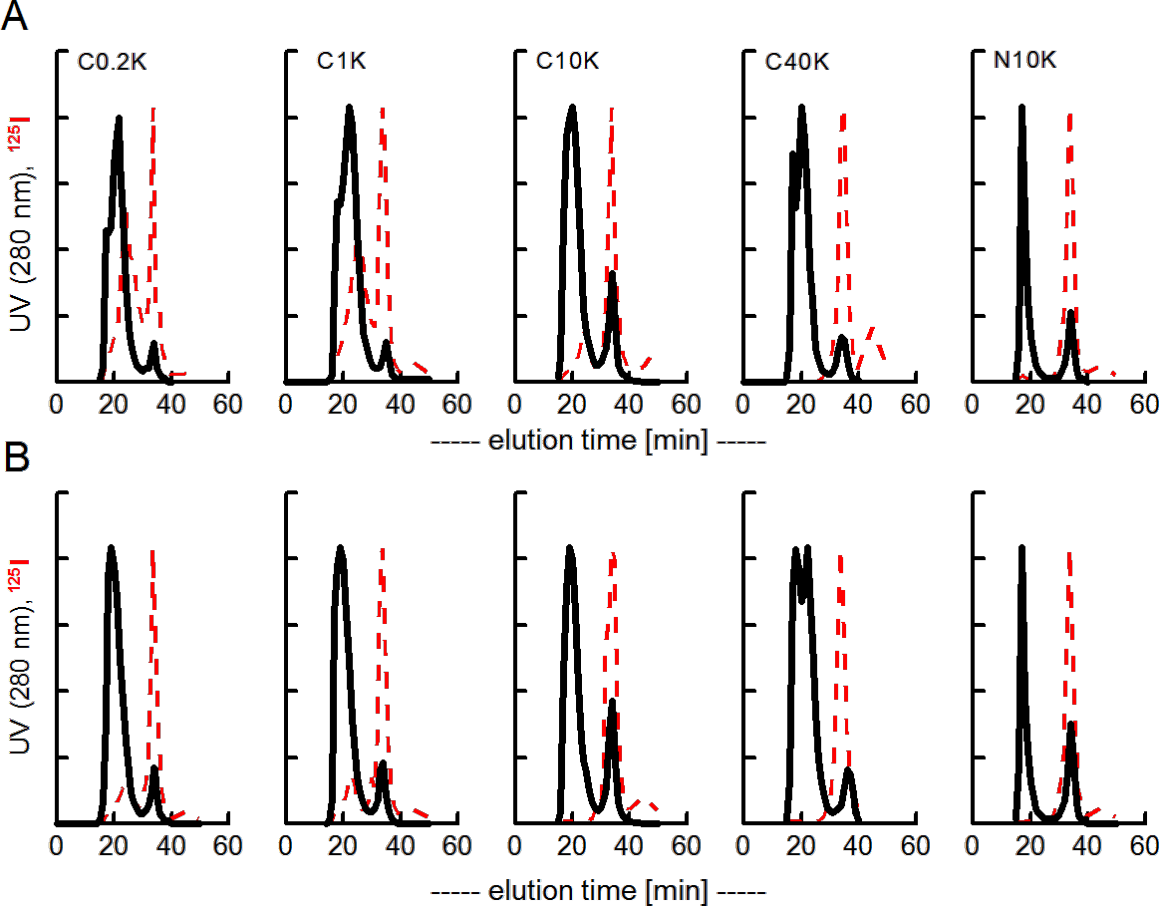
Stability of transferrin in a preformed corona on SPIOs with or without PEGylation (B). FPLC analyses of ^125^I activity in fractions. A, transferrin adsorbed; B, transferrin covalently bound. The N-PEGylated SPIOS (N10K) and the fully C-PEGylated SPIOs (C10, C40) do not adsorb ^125^I-albumin. A, initial transferrin adsorbed; B, initial transferrin covalently bound. The peak at 33 min represents proteins with the size of transferrin (DLS: 9 nm).

In a second experiment, “cold” transferrin was first incubated with different SPIOs to preform a “hard” corona and then ^125I^-labeled albumin was given as the second protein (Figure 4 and Figure 5).

It shows also in this setup that primarily adsorbed transferrin is exchanged by albumin whereas covalently bound transferrin prevents the binding of ^125^I-albumin. The remaining transferrin on the SPIOs is still functional and can bind ^59^Fe when a stable precursor (Fe-NTA) is administered to the reaction mixture (Figure 4B III).

The same results are obtained when PEGylated SPIOs are used. Albumin can partly displace adsorbed transferrin but not covalently bound. Note that the fully PEGylated species (C40K, methoxy-PEG amine used) and N10K (α,ω-bisamino-PEG) seem not to bind any protein in this experiment.

### In vivo experiments

**Figure 6:**
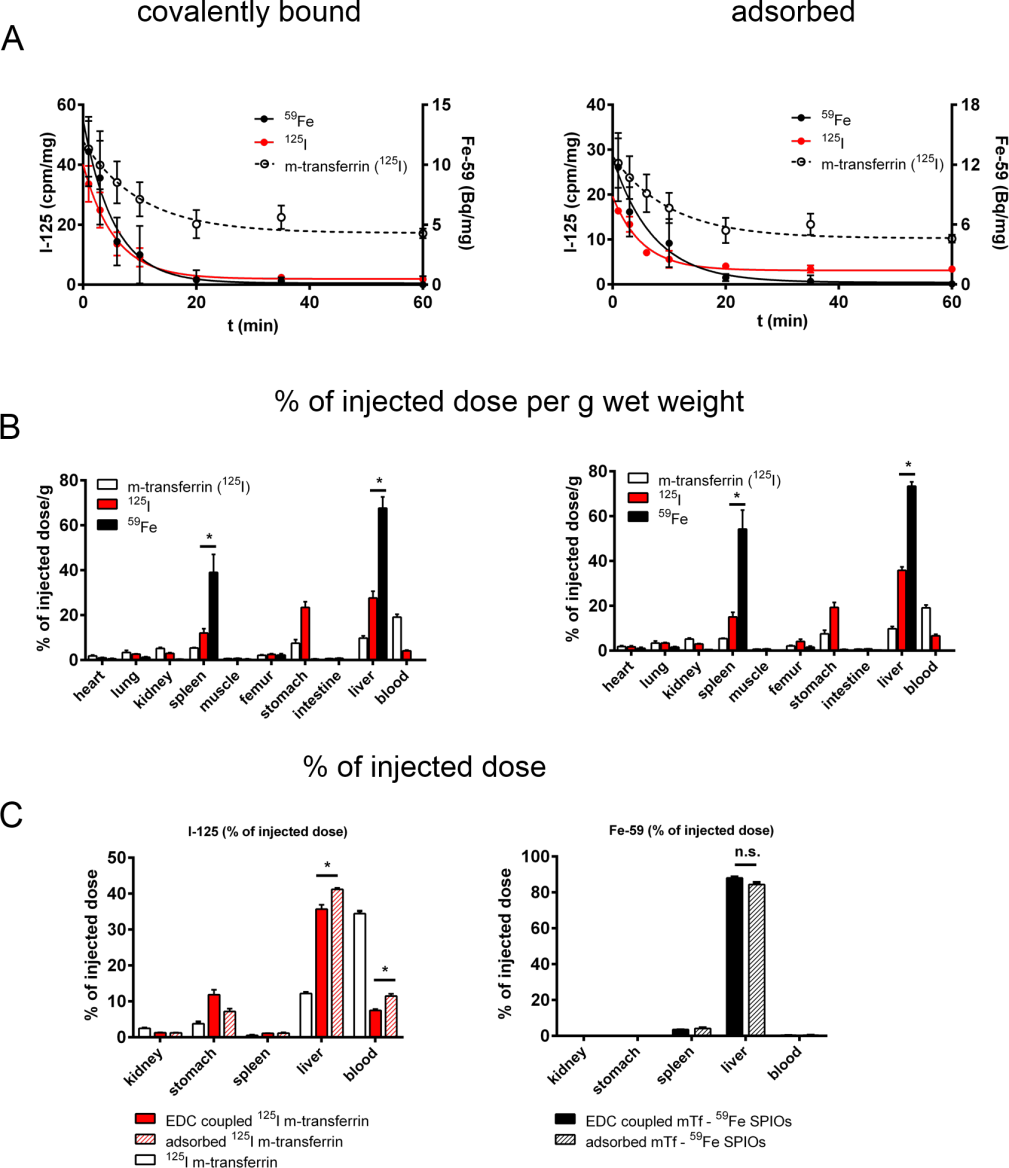
Fate of a preformed transferrin corona in vivo. A, activity of ^59^Fe and ^125^I (1–120 min) in blood; B and C, activity of ^59^Fe and ^125^I in organs 120 min after i.v. injection of ^59^Fe-SPIOs with a preformed protein corona of adsorbed (A,B right side) or covalently bound (A,B, left side) ^125^I-mouse-transferrin.

For the in vivo experiments the polymer-coated SPIOs were labelled also in the iron oxide core with ^59^Fe [27]. Two batches of ^59^Fe-labelled SPIOs were incubated with ^125^I-mouse transferrin in the presence or without EDC. Excess free transferrin was removed by filtration using a 100,000 Da filter unit. 200 µL aliquots were then injected into the tail vein of two groups of mice. ^125^I and ^59^Fe-activities were detected in blood samples in the time period between 1 min and 2 h (Figure 6A) and a very fast and synchronous removal of both labels from blood was monitored with an apparent blood-half-live of 3.6 min (^59^Fe) and 5 min (^125^I) for adsorbed and 3.8 min (both labels) for covalently bound transferrin.

After 2 h, the mice were sacrificed by blood removal and the organs were perfused with saline and measured for radioactivity (Figure 6B and C). With both preformed protein coronas, the particles were quickly removed from the blood stream and incorporated into the liver as monitored by the core label ^59^Fe which is in good agreement with results from other experiments in mice which we have performed with this specific SPIOs (data not shown). The distribution of ^125^I-transferrin from a preformed SPIO-corona was also clearly different to injected free mouse ^125^I-transferrin. However, after 120 min the ^125^I/^59^Fe-ratio clearly indicated that there was already a substantial recycling of transferrin from the liver into blood and into different tissues without any difference between adsorbed or covalently bound transferrin. This clearly indicates a fast processing of the nanoparticles within liver cells. Further experiments are needed to show which liver cells, besides Kupffer cells, are involved in the fast clearance of our NPs from blood and if the preformed transferrin corona plays a role also in a specific uptake for example in hepatocytes.

## Discussion

Many experimental techniques have been used to investigate the binding of proteins to nanoparticles and some models have been proposed to rationalize the experiments [10,32]. The most accepted view on protein corona formation is that proteins that adsorb with high affinity form a “hard” corona, do not readily desorb and show larger exchange times in the order of several hours. The “soft” corona is formed by proteins that adsorb with low affinity, are loosely bound, and are in permanent exchange with other proteins [9,28]. However, the mechanisms of protein corona formation are still debated and no existing model can fully explain it [6,10,29]. Comparing different NPs in blood plasma, it has been shown experimentally that the physico-chemical properties of a given nanoparticle determine also their individual “adsorbome” [10].

In the present study we used polymer-coated monodisperse SPIOs (11 nm core, total hydrodynamic diameter about 25 nm), as well as PEGylated variants as model nanoparticles. The iron oxide cores were labelled with ^59^Fe and we used an iodination kit to label also transferrin or albumin with ^125^I to trace the exchange of corona proteins in vitro and also in vivo in mice. These two proteins are of physiological relevance with albumin as the most abundant plasma protein and transferrin with a well understood function in iron transport and already widely used in transferrin-conjugated nanoparticles [20–21,23]. As shown in a recent metaanalysis of adsorbed corona proteins from 63 different nanoparticles in 26 studies, our chosen test proteins were also among the group of adsorbed plasma proteins which show a high abundance (>10%) in coronas from many NPs [10].

Most of the results on the composition, structure, and binding kinetics of the protein corona were so far performed ex situ and required the adequate isolation of the NPs from an artificial or physiological environment for example by differential centrifugation (DC) of size exclusion chromatography (SEC) [30–31]. These techniques are easy to perform and allow the use of modern measuring technology to analyze parameters of the protein corona. In the present study we used also 100 kDa centrifugation filters to remove unbound free protein from the nanoparticle-protein complex. It is quite clear and discussed broadly in the literature that this, as any other separation technique, has a risk of already removing loosely bound protein from the equilibrium [32]. However, for our experiments also non-removal of unbound protein would interfere with the results especially in the in vivo experiment with ^125^I-transferrin which has its own biodistribution. Our intention was less to add precise binding data on another SPIO example to the literature but more to develop a technique that would allow studying the influence of a preformed test corona also in vivo. This gives reduced amount of information on exchanging parameters of the protein corona but we can follow and quantify the consequences of a protein corona formation on the biodistribution of the particle and the test protein directly in vivo, a topic that is most relevant for any nanomedical application in the future.

We first looked in an in vitro experiment for the equilibrium binding of ^125^I-human transferrin on polymer-coated SPIOs of about 25 nm, which are negatively charged due to carboxylgroups on the surface and on PEGylated variants, which provide less negative groups or are even positively charged. As expected, the extent of transferrin binding decreased with the grade of PEGylation, in some highly PEGylated variants (C40K and N10K) obviously no protein was bound. This tunable “postsynthetic” PEGylation of a polymer coated SPIO may offer a way to design a NP which can be optimized for limited macrophage uptake but higher affinity for a target in vivo. Complete suppression of protein adsorption, however, seems not to be the right strategy because this would also diminish the desired specific binding to a cell surface receptor [33].

In the in vitro experiments, we showed that transferrin and also albumin can bind to our polymer coated-SPIO as well as to some PEGylated variants. This is in agreement with earlier findings by Jiang et al. and Röcker et al. using smaller (10 nm) FePt-particles which had a similar coating [20,34]. They found by fluorescence correlation spectroscopy that both proteins adsorb onto the particles with affinities in the micromolar range (transferrin 26 µM, albumin 5.1 µM) and created single protein monolayers (7 nm, 3.3 nm, respectively) on the nanoparticle surface.

When our preformed adsorbed ^125^I-transferrin corona was exposed to albumin, transferrin was removed from the particles and appears in the FPLC peak of unbound free protein. This finding of a sequential binding pattern of different plasma proteins with increasing binding affinities would support the so-called “Vroman effect” which has been documented for a variety of nanoparticles using different methods [9,21,30,35]. However, with other particles under different conditions it was proven that the binding of proteins can also be mostly irreversible forming a very long-lasting “hard” corona in blood [34,36]. Using sulfonate- or carboxy-modified polystyrene latex beads and fluorescence-labeled transferrin, Milani et al. found a first layer of transferrin irreversibly bound to the particles whereas a secondary or third layer is interchanging with other proteins or lipids [21]. One explanation for different results could be the size of particles in relation to the protein size. In most studies NPs >40 nm were used and thus the size of the adsorbate (here transferrin with 9 nm) is much smaller than the adsorbent. If the nanoparticle and protein are in the same size, the Langmuir adsorption formalism seems not to be valid. This was discussed by Liu et al. using small CeO_2_-particles and bovine serum albumin with similar size of 7 nm [36]. The smaller surface free energy of particles <10 nm is more and more balanced by shear forces due to Brownian motion with the consequence of detachment of proteins. The authors explained their finding with a heteroaggregation model in which a low number of SPIOs is stabilized between layers of proteins. In our FPLC study we do not see such large protein-SPIO-aggregates with our larger particle. In striking contrast to our findings, Jansch et al. incubated smaller USPIOs with plasma proteins and found no albumin in the corona and excluded a “Vroman effect” in their setting [37]. It therefore looks like that the protein corona formation of a given nanoparticle is not simply predictable and depends on the individual properties of the respective particles.

In our pilot study in vivo, we could measure an almost quantitative uptake of the preformed adsorbed transferrin corona into the liver and spleen presumably by professional phagocytes such as Kupffer cells in the liver. We found earlier by electron microscopy of murine liver tissues after i.v. injection of the polymer coated SPIO that these monodisperse iron cores are present in endosomal structures of Kupffer cells and liver sinusoidal endothelial cells [38]. These negative charged SPIOs had no preformed transferrin corona but must have been also coated by plasma proteins before liver uptake took place implicating a similar biodistribution. This confirms the stability of a corona in a recent study by Wang et al. using a cell culture model [22].

## Conclusion

This pilot study documents the importance of in vivo experiments to show the uptake and degradation of corona proteins from designed nanodevices. For this purpose, quantitative methods are needed and we show here that radiolabelling of corona proteins and the cores of NPs can be valuable tools. Clearly further studies are needed to get more insight into the properties and influence of a preformed or a natural corona formation on the biodistribution and degradation of nanoparticles in vivo. We speculate that a tunable protein corona formation could be a successful strategy to increase targeting efficiency at least for nano diagnostic using functionalized SPIOs in magnetic resonance imaging.

## Experimental

### Synthesis of nanoparticle

The superparamagnetic iron oxide nanoparticle was synthesized according to reported procedures with slight modifications [24]. In brief, a mixture of 0.178 g FeOOH (2.0 mmol), 2.26 g oleic acid (8.0 mmol) and 22 mL 1-octadecene was heated to 320 °C under nitrogen and kept at this temperature for about 80 min. The 11 nm core sized particles showed a narrow size distribution (less than 10% standard deviation) as confirmed by transmission electron microscopy (TEM).

Encapsulation with poly(maleic acid-alt-1-octadecene) (PMAOD) solution was achieved as described in [27] using a slightly modified method [25]. The polymer-coated SPIOs were stable in buffer solution for at least 12 weeks. After protein addition, no signs of aggregation were seen during the time of the experiments (1–3 days).

PEGylation was performed by incubation at room temperature overnight of SPIOs in SBB buffer pH 9 in the presence of 1 mM PEG (methoxy-PEG-amine 5000D or α,ω-bisamino PEG 10000D (Rapp Polymer, Tübingen, Germany) and variable amounts of EDC (Sigma) referring to SPIOs:EDC-ratio of 1:200 up to 1:40000 following a method as described [26]. Excess EDC and free PEG were removed by repeated ultrafiltration in 10000 Da filter units (Pall Filtersystems GmbH, Crailsheim, Germany) and redissolving in SBB.

### Radioactive labelling of proteins and SPIOs

2–5 µL aliquots of commercial ^125^I-sodium iodide solution in 0.1 M NaOH (20–50 µCi, Perkin-Elmer, Rodgau, Germany) were added to a solution of 2 mg of human (Sigma-Aldrich, Munich, Germany) or mouse transferrin (Rockland Immunochemicals Inc., Gilbertsville, PA, USA) in 1 mL sodium borate buffer pH 9.0 (SBB) together with a iodination bead (Pierce, Rockford, USA) and incubated for 15 min at room temperature. The solution was loaded onto a PD10 column and 1.8 mL of SBB was added. The iodinated protein was then eluted by additional 1.3 mL of SBB. The eluate was concentrated to the desired concentration by ultrafiltration (5000 Da filter units).

Iron oxide cores of SPIOs were labelled with ^59^Fe as described earlier [27]. In brief, aliquots of a ^59^FeCl_3_ – stock solution in 0.5 M hydrochloric acid (20–50 µCi, Perkin-Elmer, Rodgau, Germany) were lyophilized to remove water and traces of hydrochloric acid. Then earlier synthesized monodisperse oleic acid stabilized SPIOs in chloroform were added (*c* = 1–5 mg SPIOs/mL). The solution was stirred at room temperature for at least 24 h before using the SPIOs for further experiments.

### Incubation of SPIOs with proteins

^59^Fe-labeled polymer-coated SPIOs were incubated with ^125^I-labeled mouse transferrin (mTf) in the presence of EDC (1-ethyl-3-(3-dimethylaminopropyl)carbodiimide hydrochloride, Sigma-Aldrich) or in the absence of EDC. Therefore equal amounts of a 6 µM ^59^Fe-SPIO solution and 600 µM ^125^I-mTf each in 50 mM sodium borate puffer (pH 9.0) were mixed. For a covalent binding of mTf to the nanoparticle EDC dissolved in the same buffer was added to achieve a ratio of EDC molecules to nanoparticles, *c* (EDC)/*c* (NP), of 10000. To let the mouse transferrin adsorb to the particle surface buffer without EDC was added. The samples were allowed to react at room temperature for 2 h first and then at 4 °C overnight. EDC and excess free transferrin were removed by filtration using a 100 kDa centrifugal filter unit. Finally, the particle solutions were filtered through a 0.22 µm Millipore filter.

For the in vitro experiments the different PEGylated SPIOs were incubated with human transferrin and bovine albumin (Sigma) under the same conditions as described above. After incubation free protein was removed by filtration using a 100 kDa centrifugal filter unit (Amicon Ultracel-100 membrane). In a second or third step, the exchange proteins (albumin or human plasma) were added in a 3 fold excess to the initial transferrin concentration. After renewed filtration, aliquots were drawn before and after FPLC fractionation and analysed for ^125^I or ^59^Fe-activity.

### Size-exclusion-chromatography (SEC)

SEC was performed using a Superose-6 10/300 GL column (Amersham Bioscience, Munich, Germany) with buffer (10 mM tris(hydroxymethyl)aminomethane, pH 8.0; 0.15 mM NaCl, 10 mM EDTA) at a flow rate of 0.5 mL/min. Under UV 280 nm detection, fractions of 0.5 mL were collected and ^125^I and ^59^Fe were measured using a γ-counter. The column was calibrated by injecting a sample of human plasma and DLS-measurements were performed in fraction (Figure 1). Figures 2–5 represent chromatograms from single runs. However, rechromatography with samples stored at 4 °C for several days showed virtually the same results.

For iron detection, 200 µL of each fraction were treated with 50 µL of 5 M hydrochloric acid at 70 °C for 30 min. Afterwards 150 µL of a 2 M acetate buffer (pH 4.8) containing 10% ascorbic acid was added to 50 µL of each fraction, followed by 100 µL of a solution of 50 mg bathophenanthroline in 50 mL water. After 15 min, the absorption was measured at 540 nm.

### Radioactivity measurements

^59^Fe in organs or living mice was measured using the large volume Hamburg whole body radioactivity counter [39]. ^125^I was measured with an automatic gamma counter (Perkin Elmer 2470 Wizard).

### DLS measurement

Dynamic light scattering of SPIOs or proteins solutions in PBS-buffer was performed using a Malvern Zen1690.

### In vivo studies

All animal experiments were approved by the local committee for animal experiments (Behörde für Soziales, Familie, Gesundheit und Verbraucherschutz, BSG, Hamburg Tierversuchs-Nr. 34/10).

In wild type FVB mice 200 µL of either a solution containing ^59^Fe-SPIOs with adsorbed or covalently bound mouse ^125^I-transferrin were injected into the tail vein. To determine the blood half-life, 20–50 µL of blood were taken from the retroorbital venous plexus in the time period between 1 and 60 min. At the end of the experiment, the mice were anaesthetized by i.p. injection of Xylazin/Ketamine. 120 min after nanoparticle injection the torso was opened and blood was removed from the right ventricle using an EDTA-coated syringe. The right atrium was opened and PBS containing 10 units of heparin was perfused via the left ventricle for 3 min. Then, the organs (spleen, kidney, liver, etc.) were harvested, weighted and the radioactivity was determined in the fresh samples.

### Statistics

To assess statistical significance the two-tailed, unpaired Student’s t-test was performed. *P* < 0.05 was considered as significant.
